# Ecological niche differences between two polyploid cytotypes of *Saxifraga rosacea*

**DOI:** 10.1002/ajb2.1431

**Published:** 2020-02-17

**Authors:** Lucile Decanter, Guy Colling, Nora Elvinger, Starri Heiðmarsson, Diethart Matthies

**Affiliations:** ^1^ Fondation Faune‐Flore c/o Musée national d'histoire naturelle 25 rue Münster L‐2160 Luxembourg Luxembourg; ^2^ Population Biology and Evolution Musée national d'histoire naturelle 25 rue Munster L‐2160 Luxembourg Luxembourg; ^3^ The Icelandic Institute of Natural History Borgir vid Nordurslod 600 Akureyri Iceland; ^4^ Unit of Plant Ecology Department of Biology University of Marburg Karl‐von‐Frisch‐Straße 8 D‐35043 Marburg Germany

**Keywords:** Cytotype distribution, frost tolerance, niche modeling, polyploidy, population differences, reciprocal transplant experiment, Saxifragaceae, spatial segregation

## Abstract

**Premise:**

Different cytotypes of a species may differ in their morphology, phenology, physiology, and their tolerance of extreme environments. We studied the ecological niches of two subspecies of *Saxifraga rosacea* with different ploidy levels: the hexaploid Central European endemic subspecies *sponhemica* and the more widely distributed octoploid subspecies *rosacea*.

**Methods:**

For both cytotypes, we recorded local environmental conditions and mean plant trait values in populations across their areas of distribution, analyzed their distributions by niche modeling, studied their performance at two transplant sites with contrasting conditions, and experimentally tested their cold resistance.

**Results:**

Mean annual temperature was higher in hexaploid than in octoploid populations and experiments indicated that frost tolerance of the hexaploid is lower than that of the octoploid. Reproduction of octoploids from Central Europe was higher than that of hexaploids at a transplant site in subarctic Iceland, whereas the opposite was true in temperate Luxembourg, indicating adaptation of the octoploids to colder conditions. Temperature variables were also most important in niche models predicting the distribution of the two cytotypes. Genetic differences in survival among populations were larger for the octoploids than for the hexaploids in both field gardens, suggesting that greater genetic variability may contribute to the octoploid's larger distributional range.

**Conclusions:**

Our results support the hypotheses that different cytotypes may have different niches leading to spatial segregation, and that higher ploidy levels can result in a broader ecological niche and greater tolerance of more extreme conditions.

Polyploidization is a well‐known phenomenon in the evolution of angiosperms (Grant, 1981; Masterson, 1994; Otto and Whitton, 2000; Soltis et al., 2009, 2014; Rice et al., 2015). Polyploid species contain more than two sets of chromosomes and can originate through genome duplication within a species (autopolyploidy) or hybridisation between different species and subsequent genome duplication (allopolyploidy; Soltis and Soltis 1993, 1999; Comai, 2005). Polyploidization often creates new lineages that contribute to biodiversity in plants (Glennon et al., 2014). Polyploidization can also result in changes in plant morphology, phenology, physiology, and demography (Li et al., 1996; Levin, 2002; Raabová et al., 2008; Maherali et al., 2009), and it can generate individuals that exploit new niches, have a higher tolerance of extreme environments (Weiss‐Schneeweiss et al., 2013), are more resistant against herbivores (Stutz et al., 2016), or may outcompete their parent species (Leitch and Leitch, 2008). Polyploids may also be more able to colonize new habitats and can be more invasive (Richardson et al., 2000; te Beest et al., 2012). Further polyploidization events and backcrosses can lead to cytotypes with an even higher number of chromosome sets and to species with multiple cytotypes that differ in their chromosome numbers. However, maintaining large genomes also presents metabolic costs for polyploid cytotypes that can result in lower growth rates (Otto, 2007; Neiman et al., 2013; Guignard et al., 2016). Little is known about the ecological consequences of different levels of polyploidy (Brittingham et al., 2018).

A recent review concluded that co‐occurrence of different cytotypes within populations of mixed‐ploidy species is common, but spatial segregation at different scales can occur (Kolář et al., 2015). In the case of mosaic parapatry, mostly single‐cytotype populations may be spatially intermingled, or dominant cytotypes may be spatially separated with limited contact zones (large‐scale parapatry; Kolář et al., 2015). The pattern of the geographic distribution of cytotypes can provide important insights about the origin and maintenance of different ploidy levels (Baack, 2005; Rieseberg and Willis, 2007; Kolář et al., 2009; Muñoz‐Pajares et al., 2018). A random distribution of cytotypes or the frequent occurrence of mixed‐cytotype populations can indicate similar habitat requirements, whereas strong spatial segregation may indicate niche differentiation, reproductive isolation, limited dispersal, or separation of cytotypes by historical factors (see Muñoz‐Pajares et al., 2018 and references therein).

Climate is a major determinant of the distribution of plant species and is also thought to explain a large part of the spatial separation of lineages with different ploidy levels (Glennon et al., 2014; McAllister et al., 2015; Muñoz‐Pajares et al., 2018). Ecological niche modeling (Mairal et al., 2018; Muñoz‐Pajares et al., 2018) and multivariate analysis of niche variables allow a quantitative evaluation of the ecological divergences of plant lineages with different ploidy levels and also permit a statistical comparison of the niche overlap of the different taxa (Warren et al., 2008; Broennimann et al., 2012). However, the evidence for climatic or ecological niche differentiation between different cytotypes of a species is still inconclusive. While some studies have found correlations between cytotype distribution and climate variables and differences in habitat conditions between cytotypes (e.g., Raabová et al., 2008; Kolář et al., 2009; Sonnleitner et al., 2010; Ramsey, 2011; Richardson and Hanks, 2011; Mráz et al., 2012; McAllister et al., 2015; Visger et al., 2016; Muñoz‐Pajares et al., 2018), others have found evidence for shared broad‐scale climatic niches between cytotypes and no evidence for differences in climatic requirements (Godsoe et al., 2013; Glennon et al., 2014 and references therein). Ideally, the study of niche differentiation would include experiments testing the effects of environmental factors on different cytotypes, but there are not many studies involving such experiments (Ramsey, 2011; Kolář et al., 2015; McIntyre and Strauss, 2017). As polyploidization can also drive changes in phenotypic traits in natural populations (Comai, 2005; Chen and Sun, 2010), the study of phenotypic differences between different cytotypes may be important to understand their ecology (Segraves et al., 1999; Nuismer and Cunningham, 2005; Münzbergova, 2006).

Historical factors may also strongly influence the distribution of cytotypes (Stebbins, 1984; Brochmann, 2004). The current distribution of polyploids in regions affected by the ice ages may be linked to events of colonization and retreats in refugial zones of plant species, associated with the glaciation‐deglaciation periods of the recent geological past. The frequency and level of ploidy in flowering plants increase towards the poles and circum‐arctic area (Favarger, 1967; Löve and Löve, 1974; Brochmann et al., 2004). This suggests that, because of their fixed‐heterozygosity, polyploids are buffered against inbreeding and genetic drift and are more successful at colonizing deglaciated areas than their relatives with lower ploidy levels (Brochmann et al., 2004).

In the family *Saxifragaceae*, the diversity of cytotypes within a species can be complex because of multiple euploid and aneuploid polyploidization events (Soltis et al., 2007). Within the genus *Saxifraga*, the section *rosacea* has many closely related taxa that differ in their ploidy levels (Webb and Gornall, 1989). We studied the possibility of niche differentiation in two subspecies with different morphology and cytotype: the octoploid *Saxifraga rosacea* ssp. *rosacea*, and the hexaploid *S. rosacea* ssp. *sponhemica* (C.C. Gmel.) D.A. Webb. Both cytotypes are considered to be Ice Age relics (Thorn, 1960; Walter and Straka, 1970; Walisch et al., 2015) and despite occurring in similar habitat types (scree, cliffs, rock walls), the two cytotypes have distinct distribution areas without known overlapping zones (Jalas et al., 1999).

We addressed the following specific questions: (1) Do the two subspecies differ consistently in their chromosome number? (2) Do the cytotypes differ in their ecological niches, and in particular, is there evidence for a wider niche and a greater tolerance of extreme conditions of the taxon with the higher ploidy level? (3) Do plants in populations of the two cytotypes differ in their size and reproduction?

## MATERIALS AND METHODS

### Study species

*Saxifraga rosacea* Moench is a perennial plant with a fragmented distribution in Europe. Three subspecies have been distinguished (Webb and Gornall, 1989), but we studied only *S. rosacea* ssp. *rosacea* Moench and *S. rosacea* ssp. *sponhemica* (C.C.Gmel.) D.A. Webb. The third subspecies *Saxifraga rosacea* ssp. *hartii* (D. A. Webb) D.A. Webb is only known from Arranmore Island in Ireland (Chater, 1987; Webb and Gornall, 1989).

The phylogenetic relationships of *S. rosacea* s.l. are not clear. In a phylogenetic study of *Saxifraga* sec. *Saxifraga*, Vargas (2000) identified a polytomic subclade consisting of *S. rosacea*,* S. hartii*,* S. graeca*,* S. granulata* and *S. cespitosa*. More recently, Tkach et al. (2015) found *S. terektensis*,* S. cespitosa* ssp. *monticola* and *S. hypnoides* to be closely related to *S. rosacea*. The two taxa we studied were originally described as different species (s. Webb and Gornall, 1989), and in the interest of brevity, we will refer to them as *S. rosacea* and *S. sponhemica*. Chromosome numbers for *S. sponhemica* have been reported to be 46–52 in Central and Eastern Europe (Drabkova, 2000; Oberdorfer et al., 2001), whereas plants of *S. rosacea* from Clare Island and Blackhead in Western Ireland had 64 chromosomes (Philp, 1934; Webb, 1950). Preliminary results from crossing experiments indicate that reproduction of hybrids between *S. sponhemica* and *S. rosacea* is very low (Decanter, unpublished data).

**Figure 1:**
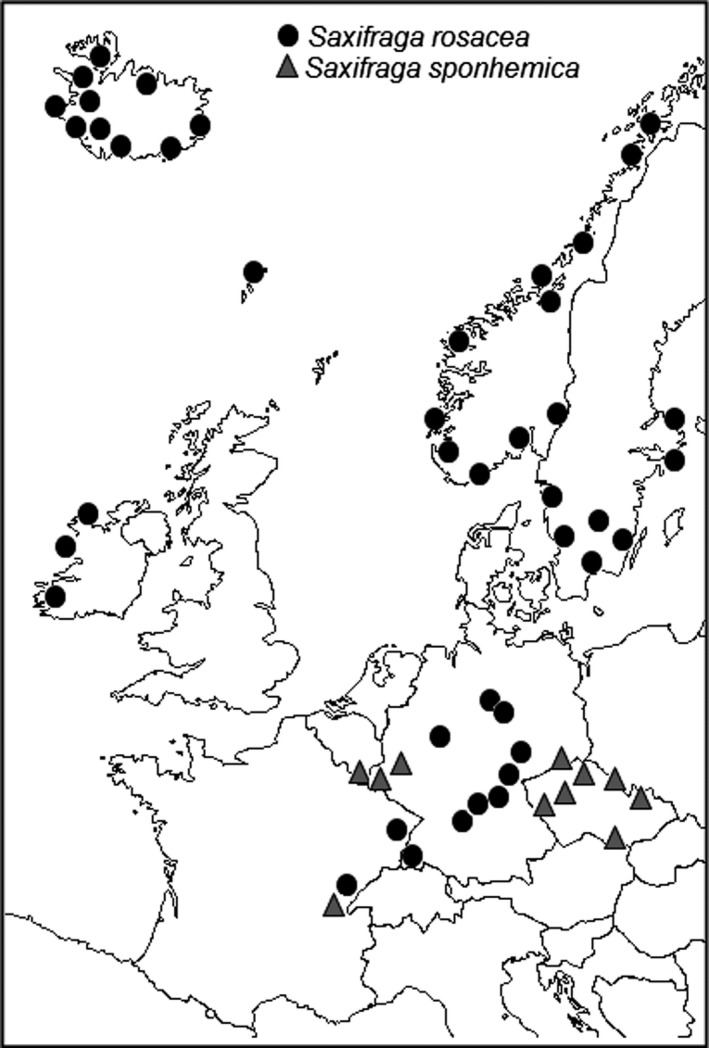
Map showing the distribution of *Saxifraga rosacea* and *Saxifraga sponhemica* (modified from GBIF 2019 and Atlas Flora Europeae; Jalas et al., 1999).

Both *S. sponhemica* and *S. rosacea* have disjunct distributions (Fig. 1). *Saxifraga sponhemica* occurs in an area extending from the Belgian Ardennes to the German Hunsrück, in the French Jura, in the Czech Republic, and in the extreme south of Poland (Jalas et al., 1999). *Saxifraga rosacea* occurs in Central and Eastern Germany, the Faroe Islands, Western Ireland, Norway, Sweden, Finland, and in Iceland (Webb and Gornall, 1989; GBIF, 2019).

Both subspecies grow as compact cushions ranging in size from 1–600 rosettes and occur in stable environments where competition is low, such as rocky substrates, screes, stony slopes, and stone walls (Webb and Gornall, 1989). The lobes of the leaves of *S. rosacea* are obtuse, acute, or shortly mucronate with hairs that are predominantly glandular, whereas the segments of the leaves of *S. sponhemica* are apiculate, narrow, and have hairs that are mostly nonglandular (Webb and Gornall, 1989). In continental Europe, both taxa flower from April to July, whereas in Iceland, *Saxifraga rosacea* flowers from June to August. If not visited by insects, the white, protandrous flowers produce hardly any seeds (Web and Gornall, 1989).

### Study sites

We selected nine populations of *S. sponhemica* spread over its area of distribution: three populations in the Czech Republic, three in the Ardennes (Belgium and Luxembourg), two in the Hunsrück region of Germany, and one in the French Jura. We selected 13 populations of *S. rosacea*: four in Iceland, four in Eastern Germany, three in Southern Germany, one in the French Jura, and one in the Vosges mountains of France (Table 1).

**Table 1:** Locations of the 22 populations of *Saxifraga rosacea* and *Saxifraga sponhemica* in which the environmental conditions, the vegetational composition, and the population structure were studied. In a number of populations additional studies were carried out.

Region	Population	Abbreviation	Altitude (m a.s.l)	Lat.	Long.	Studies
***Saxifraga rosacea*** **spp. ** ***sponhemica***						
Ardennes	Bouillon	BOU	230	49.79	5.06	T, F, Ts, Cc
	Kautenbach	KAU	277	49.95	6.01	T, F, Ts, Cc
	Robertville	ROB	474	50.45	6.10	F, Ts, Cc
Hunsrück	Frauenberg	FRA	313	49.66	7.28	T, F, Ts, Cc
	Gerolstein	GER	417	50.19	6.62	T, F, Ts, Cc
Czech Republic	Blesno	BLE	466	50.45	14.14	T, F, Ts, Cc
	Tetínské skály	TET	249	49.95	14.10	T, F, Ts, Cc
	Voskov	VOS	237	49.91	14.19	T, F, Ts, Cc
Jura	Arbois	ARB	531	46.87	5.81	T, F, Ts, Cc
***Saxifraga rosacea*** **spp. ** ***rosacea***						
East Germany	Hatzfeld	HAT	359	50.99	8.54	T, F, Ts, Cc
	Hersbruck	HERS	454	49.51	11.48	T, F, Ts, Cc
	Moschwitz	MOS	345	50.53	12.16	T, F, Ts, Cc
	Rubeland	RUB	413	51.75	10.84	T, F, Ts, Cc
South Germany	Hermentingen	HERM	659	48.19	9.21	
	Wental	WEN	622	48.73	10.01	T, F, Ts, Cc
	Wentalwieble	WENW	615	48.71	10.01	T
Iceland	Aðaldalshraun	ADA	4	65.93	–17.49	T, Ts, Cc
	Road 427	R427	39	63.85	–22.20	F, Ts, Cc
	Skógafoss	SKO	79	63.53	–19.51	T, F, Ts, Cc
	Svart	SVA	52	63.88	–22.41	T, F, Ts, Cc
Vosges	Hartmannswillerkopf	HAR	909	47.85	7.16	T, F, Ts, Cc
Jura	Baume‐les‐Messieurs	BAU	416	46.69	5.64	T, F, Ts, Cc

Note

F = samples selected for the frost tolerance experiment, T = temperature data logger placed at site, Ts = rosettes from population planted into transplant sites, Cc = number of chromosomes counted.

### Chromosome counts

Chromosomes were counted for plants from all the study populations (excluding the populations from Hermentingen [HERM] and Wentalwieble [WENW], Germany) using the protocol of Inceer and Hayirlioglu‐Ayaz (2007). In summer 2012, one rosette from each of at least 20 cushions (plants) was collected in each of the populations. The rosettes were placed onto soaked peat pellets (Jiffy, Jiffy Group, Kristiansand, Norway) to grow roots, and in spring 2013, the rosettes were planted into pots. The plants were kept in the garden of the National Museum of Natural History of Luxembourg. In autumn 2014, roots of three individuals per population were sampled, gently cleaned with ddH_2_O taking care not to damage the rootlets, and placed for 3 h in a 0.05% solution of colchicine. Once wiped, the roots were placed in a 3:1 mixture of ethanol/acetic acid for 2 h at room temperature. After rinsing with ddH_2_O, the rootlets were placed together with drop of a 1% aceto‐orcein solution between a slide and a cover glass, and gently squashed. Chromosomes were counted under a microscope with a magnification of 1000X (Motic serie BA210 Digital, Hong Kong, People's Republic of China).

### Habitat characterization

In June 2012, we randomly selected plots of 1 m × 1 m in which *Saxifraga* was present in the study populations. In most populations, five plots were selected, but in the populations Arbois, Hermentingen, Aðaldalshraun, and Skógafoss only two to four plots could be established because parts of the populations were difficult to access. In each plot, we recorded the cover of each vascular species and the total cover of bryophytes, litter, and bare soil. At each site, we took a rock sample, and recorded the habitat type (e.g., rock wall, scree), slope, and altitude. The content of SiO_2_, Al_2_O_3_, Fe_2_O_3_, and CaO in the rock samples were analyzed by x‐ray fluorescence spectrometry (ARL PERFORM‐X 9400 Sequential XRF XP, Thermo Scientific, Waltham, Massachusetts, USA). Loss on ignition was determined by preparing samples (0.5 g) on glass disks with lithium metaborate, finely grinding them, and incinerating them for 2 h at 1000°C.

In July 2013, we recorded canopy openness in the study populations by taking a hemispherical photograph at a height of 1 m above one randomly selected *Saxifraga* cushion near the center of each population with a camera (Canon EOS 5D, Canon Inc., Ota City, Tokio, Japan) equipped with a Fisheye lens (EF 8–15 mm f/4L). The pictures were processed with the GapLight Analyzer software (Version 2.0, Frazer et al., 1999). From 2012–2015, we used data loggers (Tinytag Transit 2 TG‐4080 and Tinytag Plus 2 TGP‐4500, Gemini Data Loggers, West Sussex, United Kingdom) to record local temperatures at 6:00, 12:00, 18:00, and 24:00 each day in all populations except Robertville [ROB] (Belgium) and Hermentingen [HERM] (Germany). Data loggers were placed at ground level in a location that avoided direct exposition to the sun. Growing degree days (GDDs)—a measure of heat accumulation (McMaster and Wilhelm, 1997)—were calculated daily as

$$\text{GDD}=\frac{\mathrm{Tmax}+\mathrm{Tmin}}{2}-Tbase$$

with Tmax as maximum temperature registered and Tmin as minimum temperature registered. We chose 5°C as Tbase (threshold base temperature) because both cytotypes occur in cold areas. To obtain the GDDs per population, we first averaged the GDD values for each day over the measuring period and then summed the mean daily values per year. We also obtained four fundamental bioclimatic variables for each study site at a grid size of 0.86 km² (30 arcsec) from the WorldClim v2.0 database (Fick and Hijmans, 2017): mean annual temperature, maximum temperature of the warmest month, minimum temperature of the coldest month, and precipitation per year.

### Population size structure

In July 2013, we randomly selected five plots of 1 m × 1 m in each population, except for Arbois, Hermentingen, and Skógafoss, where only 2–3 plots could be placed because of problems in accessing the plants. We recorded in each plot the maximum length and width of all *Saxifraga* cushions and the number of inflorescences per cushion and calculated the mean number of inflorescences per flowering plant. To estimate the mean number of flowers per inflorescence, we counted the number of flowers on five randomly selected inflorescences per cushion. We calculated the area of each cushion by multiplying the maximum length and width of the rosette and then calculated the density of inflorescences per square centimeter of cushion. We distinguished three classes of plants based on their status and size (cushion area): (1) single vegetative rosettes, (2) medium sized cushions (one flowering rosette, or more than one rosette and cushion area <46 cm^2^), and (3) large cushions (>46 cm^2^). The value of 46 cm^2^ is the geometric mean of the cushion area of 1476 observed plants (excluding single vegetative rosettes).

### Frost tolerance experiment

We tested the frost tolerance of *S. rosacea* and *S. sponhemica* by measuring leaf electrolyte leakage using a protocol adapted from Lindén (2002). Experimental plants had been grown clonally from rosettes sampled from different mother plants in 19 populations (10 of *S. rosacea*, 9 of *S. sponhemica*) in 2012 (Table 1). The experiment was conducted in February 2014 to take advantage of the acclimatization of the plants to winter temperatures in the botanical garden of the Natural History Museum in Luxembourg. We collected three pairs of leaves from each of five mother plants per population. Each pair of leaves was cleaned and placed for 24 hours pairwise into a small plastic bag filled with ddH_2_O to maximize leaf turgor. The three leaf pairs from each maternal plant were then briefly placed on absorbing paper to remove water on the leaf surface, and each pair was sealed in a plastic bag and subjected to one of the three temperatures in a dark climatic chamber: –10°C, –20°C, or +23°C as control. After 24 hours, we collected 20 circular tissue samples (0.5 mm diameter) from each leaf pair with a leaf puncher (Harris Uni‐Core 0.5, Sigma‐Aldrich, St. Louis, Missouri, USA), transferred them into Eppendorf tubes with 5 mL of ddH_2_O, and stirred them (250 rpm) at room temperature for 24 h. Conductivity was then measured with a conductivity meter (SensION+ EC71, Hach, Tomball, Texas, USA). The leaf samples in the Eppendorf tubes were then placed for 20 min in a water bath at 92°C, stirred again (250 rpm) at room temperature for 24 hours, and conductivity was measured again for each leaf pair. The electrolyte leakage ratio was calculated as the ratio between the first and the second conductivity measure and expressed as a percentage.

### Test of local adaptation

We grew plants of each cytotype at two common transplant sites, one in Luxembourg (49°57′54″N, 5°58′09″E, 297 m a.s.1.) and a second one in northern Iceland (65°44′27″N, 17°59′02″W, 508 m a.s.1.). The two transplant sites were located near extant populations of *S. sponhemica* and *S. rosacea,* respectively, but beyond the range of the other cytotype. The transplant site in Luxembourg was established in an abandoned slate quarry near Merkholtz. All existing plant cover was removed, and the area was covered with a TYPAR geotextile (DuPont de Nemours, Inc., Wilmington, Delaware, USA) to avoid competition by local vegetation. The geotextile was covered with a 10 cm layer of a local schist and soil mixture. The transplant site in northern Iceland was set up on top of a flat mountain ridge (540 m) near Akureyri covered by a very sparse and eroded heathland vegetation. The site was fenced to avoid grazing by sheep and horses. Temperature data were recorded for 24 months at each site with data loggers (Tinytag Transit 2 TG‐4080; Gemini Data Loggers Ltd., Chichester, West Sussex, United Kingdom) placed at a soil depth of 5 cm starting in August 2013 (Iceland) and October 2013 (Luxembourg).

In June 2013, four newly formed rosettes were sampled from five randomly selected mother plants from each of the populations used for the frost tolerance experiment (Table 1). Each sampled rosette was planted into a peat pellet (38 mm) (Jiffy, Jiffy Group, Zwijndrecht, The Netherlands) and grown in the botanical garden of the Natural History Museum in Luxembourg. We planted two daughter rosettes from each mother plant (genotype) into each of the two transplant sites at the end of July 2013 (Iceland) and mid‐September 2013 (Luxembourg). The daughter rosettes were planted with their peat pellets into the local soil. In total, 200 plants originating from 11 *S. rosacea* and 9 *S. sponhemica* populations were planted per transplant site. To avoid intraspecific competition, we randomly planted 20 plants separated by a distance of 50 cm on 10 transects. The distance between transects was 1 m. In July (Iceland) and August (Luxembourg) 2014 and 2015, we recorded survival, size, and flowering of the plants. Plant size was recorded as the largest diameter of a cushion. Reproductive characteristics were recorded by counting the total number of inflorescences per cushion.

### Environmental niche modeling and niche overlap

Environmental niche modeling was performed with MaxEnt software (Phillips et al., 2006) with default parameters, except for number of replicates (10), and maximum number of iterations (5000). We used the area under the curve statistic (AUC) to evaluate model accuracy. We used all 19 bioclimatic variables available from the WorldClim v2.0 database (Fick and Hijmans, 2017) in a grid size of 0.86 km² (30 arcsec) for tests run separately for both taxa. Based on the relative contribution of each bioclimatic variable to the final model, we selected the following variables for the niche modeling: mean diurnal temperature range, temperature seasonality, minimum temperature of the coldest month, temperature annual range, mean temperature of the wettest quarter, mean temperature of the driest quarter, mean temperature of the warmest quarter, mean temperature of the coldest quarter, precipitation of the wettest month, and precipitation of the warmest quarter. To assess the relative importance of the various climatic variables, we used their importance values and the contribution of a variable to the model (Phillips et al., 2006).

Overlap between niches of *S. sponhemica* and *S. rosacea* was evaluated with Schoener's D using ENMTools (Warren et al., 2008, 2010). Identity of the two niches was also tested with ENMTools using 100 replicates. We used an extended data set of 39 populations of *S. sponhemica* and 193 populations of *S. rosacea* for the niche modeling (Appendix S1). Occurrence data were extracted from GBIF (GBIF.org, 10.8.2018 and 31.8.2018/ GBIF occurrence downloads https://doi.org/10.15468/dl.kpjvnr; https://doi.org/10.15468/dl.ifynrf; https://doi.org/10.15468/dl.nediim; https://doi.org/10.15468/dl.nhrrzt) and herbarium specimens (Herbaria LUX and ICEL). Final niche model maps were made with QGIS3 (QGIS Development Team, 2019) based on the average niche models of 10 replicate Maxent runs calculated separately for the two taxa.

### Statistical analysis

We assessed differences in climatic variables (data from local data loggers and the WorldClim database), environmental characteristics, rock composition, and population characteristics among Icelandic and Central European populations of *S. rosacea* and *S. sponhemica* by analysis of variance (ANOVA) and Tukey tests. Differences in community composition among the populations of the two taxa were studied by conducting a nested canonical correspondence analysis (CCA) of log‐transformed values of the cover of co‐occurring vascular plants and bryophytes using the identity of the *Saxifraga* taxon present and of population as explanatory variables. The effects of taxon identity on community composition were tested against the variation among populations, while the effects of population identity were tested against the variation among plots. We also used CCA to compare the vegetation of Icelandic and continental *S. rosacea* sites.

We distinguished three types of populations for the analyses of frost tolerance and transplant sites: populations of *S. sponhemica*, Central European populations of *S. rosacea*, and Icelandic populations of *S. rosacea*. The effects of population type, population as a random factor nested within population type, and frost treatment were investigated by nested ANOVA. To disentangle the effects of differences between the two cytotypes in the same region from regional adaptation to the conditions in Iceland, we partitioned the effect of population type into two orthogonal contrasts between (1) Icelandic populations of *S. rosacea* vs. Central European populations of *S. rosacea* and *S. sponhemica* and (2) Central European populations of *S. rosacea* vs. those of *S. sponhemica* in the same region. Of interest were the interactions between the two contrasts and frost treatment, which according to the rules for analyzing mixed models, were tested against the frost treatment by population interaction (Zar, 2010).

We used nested ANOVA (for continuous variables) or deviance (for binomial variables) to test for local adaptation. Population of origin and maternal genotype were treated as random factors. We tested for the effect of population type by means of the same two orthogonal contrasts we used for analysis of frost tolerance. We also tested the effect of the transplant site against the population by site interaction, the effects of the contrasts against the variation among populations, the effect of population against the variation among genotypes, and the effects of the interactions of the site with the two contrasts against the site by population interaction.

Variables were log transformed if necessary to obtain normally distributed residuals and homoscedasticity. Vegetation analyses were carried out with R for Windows (Version 3.5.1), and the Vegan package v2.5‐4 (Oksanen et al., 2019). All other statistical analyses were performed using IBM‐SPSS 25.0 (IBM Corp. 2017).

## RESULTS

### Ploidy differences and habitat characteristics

All studied *S. rosacea* plants were octoploid (8*x* = 64), while nearly all *S. sponhemica* plants were hexaploid (6*x* = 48). One *S. sponhemica* plant from Kautenbach [KAU] (Luxembourg) and one from Robertville [ROB] (Belgium) had 52 chromosomes.

**Figure 2:**
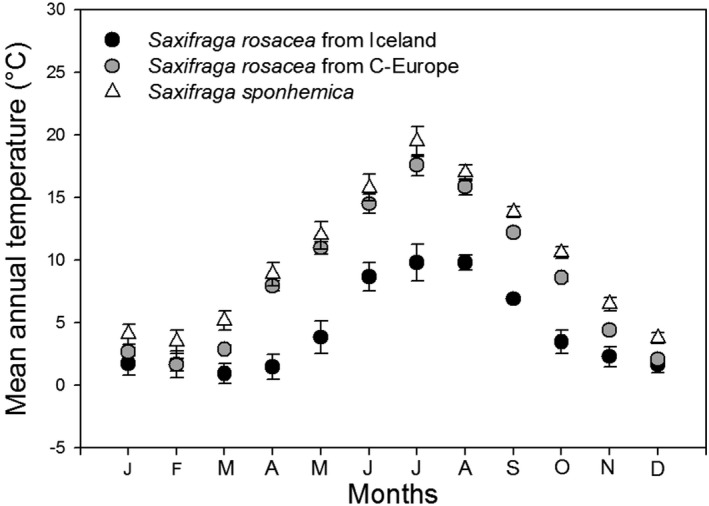
Variation in mean temperature per month among sites with *Saxifraga rosacea* in Iceland (*n* = 3), sites with *S. rosacea* in Central Europe (*n* = 8) and sites with *Saxifraga sponhemica* (*n* = 8) as measured by data loggers at the sites. Means ± 1 SE.

The mean monthly temperatures, as measured at the sites by data loggers, were lower at the Icelandic sites with *S. rosacea* than at the Central European sites with *S. rosacea* and *S. sponhemica* in nearly all months (Fig. 2), resulting in a significantly lower mean annual temperature at the Icelandic sites (Table 2). Moreover, mean monthly temperatures and mean annual temperatures in the Central European populations of *S. rosacea* were lower than in sites with *S. sponhemica*. This suggests a requirement for higher temperatures for *S. sponhemica*. Most other climatic variables differed significantly only between the Icelandic populations of *S. rosacea* and the Central European populations of both subspecies (Table 2). Icelandic populations of *S. rosacea* occurred at lower elevations and on slopes that were on average less steep than those of the Central European populations of both subspecies (Table 2). The rocks at the *S. rosacea* sites in Iceland contained more Si, Al, and Fe, and less Ca and organic material than those at the Central European sites. In contrast, there were no significant differences between the rocks on which *S. sponhemica* and *S. rosacea* grow in Central Europe and no clear differences in canopy openness among the three population types.

**Table 2:** Environmental conditions in Icelandic and Central European populations of *Saxifraga rosacea* and in *Saxifraga sponhemica* populations.

	*Saxifraga rosacea*		*Saxifraga sponhemica*	*F*
	Iceland	Central Europe	Central Europe	
**Data from WorldClim**				
Annual temperature (°C)	4.45^A^	7.86^B^	8.50^B^	44.75***
Max. temperature of warmest month (°C)	12.93^A^	21.92^B^	22.27^B^	68.27***
Min. temperature of coldest month (°C)	–2.35	–3.69	–2.97	2.19
Precipitation (mm)	1048.00	846.11	792.78	1.74
**Data from local data loggers**				
Annual temperature (°C)	4.35^A^	8.43^B^	10.28^C^	44.89***
Summer temperature (°C)	9.42^A^	15.98^B^	18.01^B^	19.07***
Maximum temperature (°C)	18.70^A^	36.46^B^	38.32^B^	3.72*
Minimum temperature (°C)	–4.91	–2.96	–4.77	0.82
Growing degree days	514.87^A^	1762.19^B^	2272.74^B^	28.84***
**Other characteristics**				
Slope (°)	22.50^A^	53.56^B^	54.67^B^	3.71*
Altitude (m)	43.50^A^	532.44^B^	354.89^B^	16.45***
Canopy openness (%)	53.67	37.50	37.83	1.24
SiO_2_ (%)	52.53^A^	17.92^B^	40.22^AB^	3.61*
Al_2_O_3_ (%)	15.17^A^	4.45^B^	10.42^AB^	4.69*
Fe_2_O_3_ (%)	11.81^A^	3.36^B^	7.65^AB^	3.85*
CaO (%)	8.89	32.41	17.82	2.29
Loss on ignition (%)	2.02^A^	30.35^B^	14.02^AB^	4.28*

Notes

*F*–test for differences between the three regions: *, *P* < 0.05; ***, *P* < 0.001. Mean values with different superscript letters are significantly different (Tukey test, *P* < 0.05).

The CCA provided no evidence for differences between the composition of the plant communities at sites with *S. sponhemica* and *S. rosacea* (*F*_1,20_ = 1.05, *P* = 0.41). However, both the individual populations of *S. sponhemica* (*F*_8,32_ = 1.79, *P* < 0.001) and those of *S. rosacea* (*F*_12,45_ = 2.05, *P* < 0.001) varied strongly in their plant communities. The vegetation of sites with *S. rosacea* in Iceland differed from that of the continental sites (*F*_1,11_ = 1.81, *P* < 0.002). The cover of vascular plant species was generally low. *Geranium robertianum* was the only plant species common to most sites of both cytotypes (present in all sites except at Tetínské skály [TET] (Czech Republic), Wental [WEN] (Germany), and in Iceland). The cover of bryophytes was low except in one Icelandic population (R427) where *Racomitrium lanuginosum* was a dominant species. The median number of species including bryophytes was 23.5 species per 5 m². In total, 228 species of vascular plants and bryophytes were co‐occurring with the two cytotypes (164 species with *S. rosacea* and 124 species with *S. sponhemica*).

### Population structure

Cushions in the Icelandic *S. rosacea* populations were very small (Table 3). The mean cushion area did not differ between both cytotypes in Central Europe, but was more than six times greater than in the Icelandic *S. rosacea* populations. Inflorescences of *S. rosacea* produced more flowers than those of *S. sponhemica* in the Central European populations and also more than those of *S. rosacea* in Iceland. The population structure of the Icelandic populations differed strongly from that of the Central European populations, because Icelandic populations had hardly any large cushions, a much higher proportion of medium‐sized plants, and more vegetative rosettes (Table 3).

**Table 3:** Characteristics of Icelandic and Central European populations of *Saxifraga rosacea* and populations of *Saxifraga sponhemica*.

Population characteristic	*Saxifraga rosacea*		*Saxifraga sponhemica*		
	Iceland	Central Europe	Central Europe	*F*	*P*–value
Cushion area (cm²)	5.9^A^	46.2^B^	39.6^B^	17.2	<0.001
Mean number of inflorescences per cm^2^ of cushion	0.14^A^	0.04^B^	0.06^B^	11.4	<0.001
Mean number of flowers per inflorescence	1.80^A^	4.48^B^	3.11^A^	12.5	<0.001
Proportion of single vegetative rosettes (%)	17.1^A^	4.0^B^	8.6^AB^	4.0	0.035
Proportion of medium cushions (%)	81.7^A^	43.4^B^	38.1^B^	18.7	<0.001
Proportion of large cushions (%)	1.3^A^	52.6^B^	53.2^B^	26.6	<0.001

Note

**, *P* < 0.01; ***, *P* < 0.001. Mean values with different superscript letters are significantly different (Tukey test, *P* < 0.05). Cushion area was log‐transformed prior to analysis. Back‐transformed means are given.

### Frost tolerance and response to environment at the transplant sites

**Figure 3:**
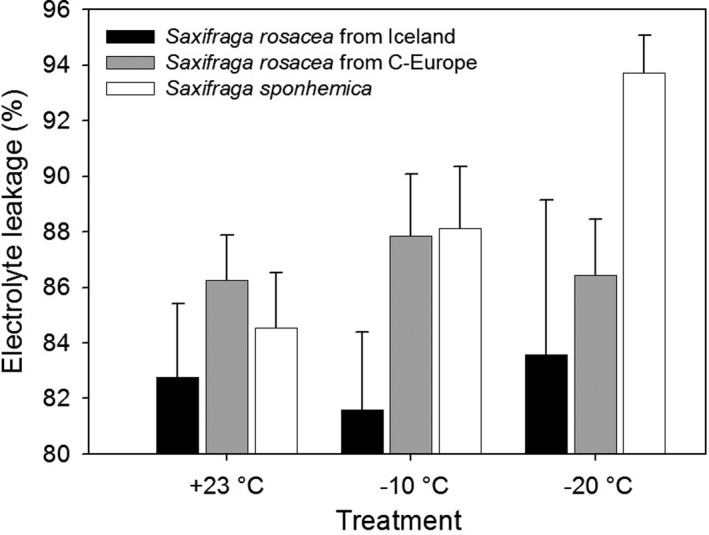
Mean (± 1 SE) electrolyte leakage ratio at three temperatures for leaves of *Saxifraga rosacea* from Iceland and Central Europe and leaves of *Saxifraga sponhemica*.

Electrolyte leakage of the leaves of *S. rosacea* from Iceland was lower than that of the leaves from Central European populations of *S. rosacea* and *S. sponhemic*a (*F*_1,16_ = 5.17, *P* = 0.037; Fig. 3), especially at frost temperatures indicating higher frost tolerance. Electrolyte leakage of leaves of *S. sponhemica* from Central European populations was much higher at –20°C than that of leaves of *S. rosacea* from the same region (*F*_2,32_ = 3.19, *P* = 0.055; Fig. 3), indicating lower tolerance of strong frost of the hexaploid *S. sponhemica*.

**Figure 4:**
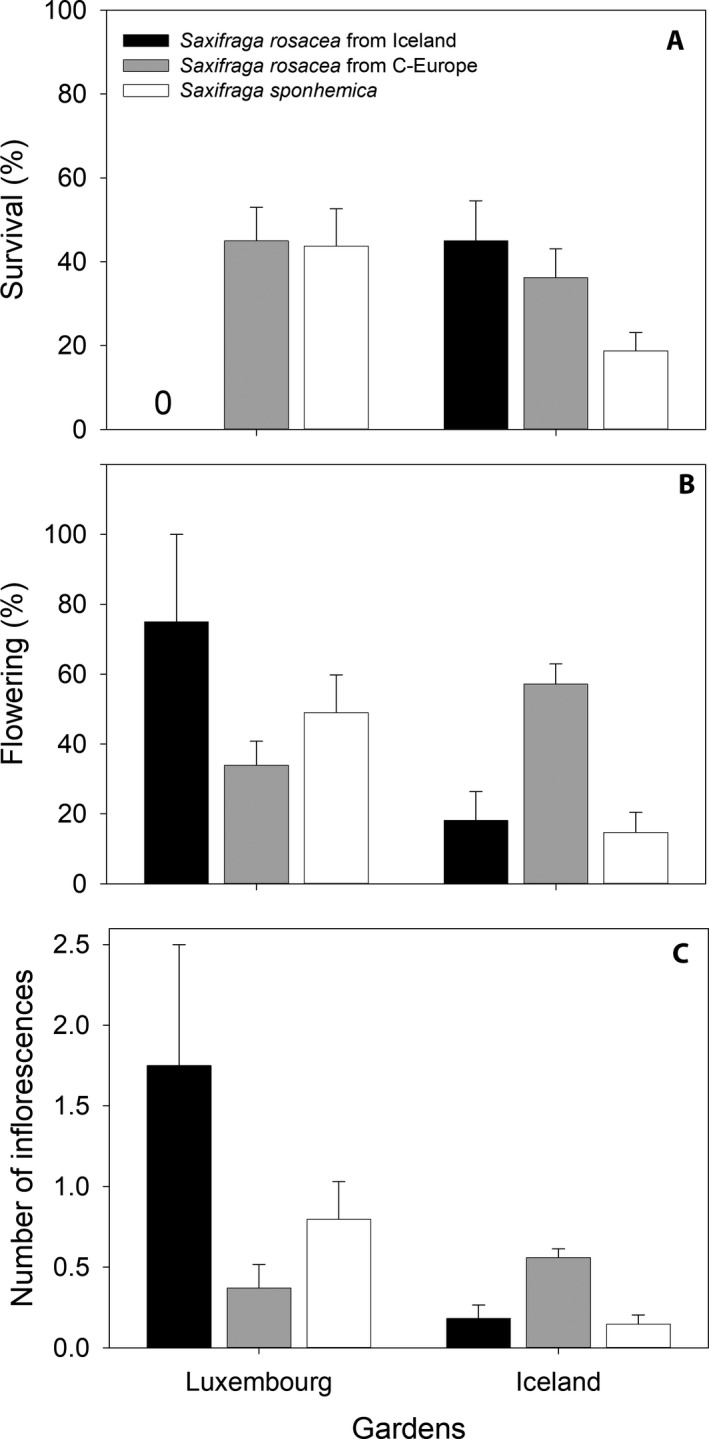
Fitness measures (mean ± 1 SE) for *Saxifraga rosacea* from Iceland and Central Europe, and *S. sponhemica* at the transplant sites in Luxembourg and Iceland: (A) The probability of survival from 2013–2015, (B) the probability of flowering in 2014, and (C) the number of inflorescences per cushion in 2014.

**Figure 5:**
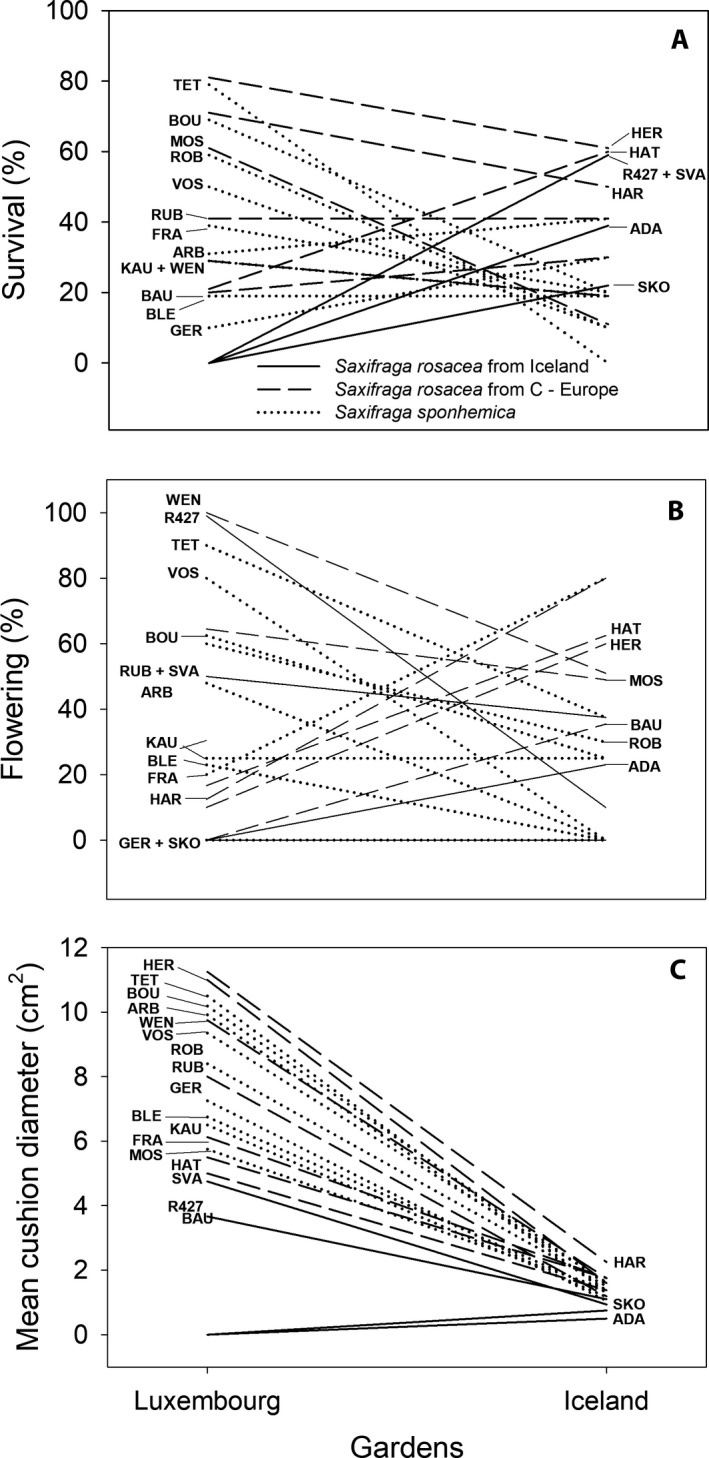
Reaction norms of four Icelandic and seven Central European *S. rosacea* populations, and nine *S. sponhemica* populations for (A) the probability of survival from 2013–2015, (B) the proportion of flowering individuals in 2014, and (C) the diameter of the cushions in 2014 at the two transplant sites.

Mean annual temperatures at the transplant sites were 2.5°C in Iceland and 11.9°C in Luxembourg. The site conditions strongly influenced all growth and reproductive characteristics. Survival until 2015 in Luxembourg was much lower (zero) for Icelandic *S. rosacea* than for both Central European population types*,* whereas in Iceland survival of the Icelandic *S. rosacea* was higher than that of both cytotypes from Central Europe (Fig. 4A, Table 4), indicating local adaptation of the Icelandic and Central European populations. However, octoploid and hexaploid cytotypes from Central Europe did not differ in their response to the two environments. The various populations of the three types varied in their reaction to the two transplant sites (Fig. 5A), indicating genetic variation. Genetic differences in survival were larger among populations of the octoploid *S. rosacea* (Icelandic and Central European populations together) than for the hexaploid *S. sponhemica* at both transplant sites. In summer 2018, when all plants had to be removed from the Icelandic site to avoid the potential spread of nonnative genotypes, only 9.5% of all plants had survived. Out of the 19 surviving plants, 16 plants belonged to the cytotype *S. rosacea* (14 Central European and two plants from Iceland). The three surviving *S. sponhemica* plants all originated from one Czech population (Voskov).

**Table 4:** Analyses of deviance for the effects of the two transplant sites, the contrast between the Iceland populations of *S. rosacea*, and the Central European populations of *S. rosacea* and *S. sponhemica*, the contrast between the Central European populations of *S. rosacea* and *S. sponhemica*, the population of origin, and genotype on survival from 2013–2014, and from 2013–2015, and flowering of the *Saxifraga* plants that survived until 2014.

	*df*	Survival		Flowering	
		2013–2014	2013–2015	2014	
		*F*	*F*	*df*	*F*
Site	1	5.5*	0.4	1	1.6
Iceland vs. C‐Europe populations	1	4.7*	3.8+	1	0.5
C‐Europe *S. rosacea* vs. *S. sponhemica*	1	0.5	2.1	1	1.9
Population	17	2.0*	1.2	17	1.5
Genotype	80	1.3+	1.2	71	1.1
Site × (Iceland vs. C‐Europe populations)	1	23.3***	19.0***	1	1.5
Site × (C‐Europe *S. rosacea* vs. *S. sponhemica*)	1	0.8	0.9	1	22.1***
Site × population	17	1.1	2.5**	15	1.8+
Site × genotype	80	1.1	1.1	38	1.0

Note

Quasi–*F* values are given. +, *P* < 0.1; *, *P* < 0.05; **, *P* < 0.01; ***, *P* < 0.001.

The two Central European population types responded differently in their reproduction to the contrasting environments (Table 4). A higher proportion of the plants of the octoploid *S. rosacea* than of the hexaploid *S. sponhemica* flowered in Iceland in 2014, whereas more *S. sponhemica* plants flowered in Luxembourg (Fig. 4B). Nearly all populations of *S. sponhemica* flowered less in Iceland than in Luxembourg, whereas both the Icelandic and Central European populations of *S. rosacea* strongly varied in their response to the two sites (Fig. 5B). At the Luxembourg site, variation among the populations of the octoploid was higher than that of the hexaploid indicating greater genetic viability, whereas in Icelandic variation was similar. Reproduction, as measured by the number of inflorescences produced by the flowering plants in 2014, was higher for the few surviving plants from the Icelandic populations at the site in Luxembourg than for the Central European populations (Table 5, Fig. 4C). However, all the plants from Iceland grown in Luxembourg died before the next year. Central European plants of the octoploid *S. rosacea* produced more inflorescences under the cold conditions in Iceland than plants of the hexaploid *S. sponhemica*, while the reproductive success of *S. sponhemica* was higher than that of the *S. rosacea* plants from the same region at the warmer site in Luxembourg, indicating contrasting responses of the two cytotypes to the two environments.

**Table 5:** Analyses of variance for the effects of the two transplant sites, the contrast between the Iceland populations of *S. rosacea*, and the Central European populations of *S. rosacea* and *S. sponhemica*, the contrast between the Central European populations of *S. rosacea* and *S. sponhemica*, the population of origin, and genotype on plant size, and the number of inflorescences per cushion of the two *Saxifraga* subspecies in 2014.

Source of variation	*df*	Cushion diameter	Number of inflorescences
		*F*	*F*
Site	1	182.4***	7.3*
Iceland vs. C‐Europe populations	1	3.0+	0.1
C‐Europe *S. rosacea* vs. *S. sponhemica*	1	<0.1	<0.1
Population	17	2.0*	0.9
Genotype	71	1.3	2.9***
Site × (Iceland vs. C‐Europe populations)	1	3.3+	5.1*
Site × (C‐Europe *S. rosacea* vs. *S. sponhemica*)	1	0.6	18.6***
Site × population	15	1.6	0.5
Site × genotype	38	1.5+	4.7***

Note

+, *P* < 0.1; *, *P* < 0.05; **, *P* < 0.01; ***, *P* < 0.001.

Overall, cushion diameter was much larger in Luxembourg (mean 2014: 7.8 ± 0.55 cm, *n* = 18) than in Iceland (mean 2014: 1.4 ± 0.09 cm, *n* = 20) after one year of growth (Table 5). Mean plant size did not differ between Central European populations of the two cytotypes and the two cytotypes did not react differently to the conditions at the sites. However, plants from Icelandic populations were smaller than those from both cytotypes from Central Europe and this difference was particularly pronounced at the Luxembourg site (4.2 ± 0.54 cm vs. 8.2 ± 0.94 cm diameter), indicating local adaptation. At the site in Luxembourg, variation in the growth of plants among the individual populations from Central Europe was large, whereas Icelandic *S. rosacea* were all small (Fig. 5C), indicating lower plasticity and a reduced ability to profit from the warmer conditions at the Luxembourg site. In contrast, in Iceland, variation in the growth of the various populations of both cytotypes was rather low.

### Environmental niche modeling and niche overlap

**Figure 6:**
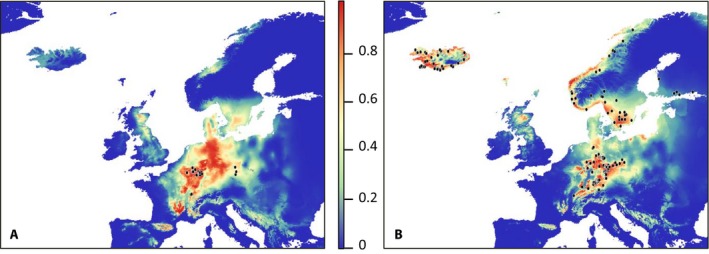
Probability of occurrence of (A) *Saxifraga sponhemica* and (B) *Saxifraga rosacea* according to Maxent niche models based on 10 climatic variables (see text for details). Black dots represent the populations used for the niche models (see Appendix S1). Blue indicates unsuitable habitats, and red colors indicate highly suitable habitats.

The estimated niche models using 10 climatic variables had very high area under the curve (AUC) scores for *S. sponhemica* (0.977 ± 0.013) and *S. rosacea* (mean 0.959 ± 0.013), indicating negligible rates of false negative and false positive suitability predictions. Nearly all populations of *S. sponhemica* and *S. rosacea* were situated in areas with high predicted probabilities (Fig. 6A, B). A niche overlap between the two taxa of 56.6% (Schoener's D) was significantly lower than expected by chance in the niche identity test (83.7% ± 1.9%, *P* < 0.001). In the final model, temperature annual range had the highest contribution to the model for populations of both *S. rosacea* (49.4%) and *S. sponhemica* (41.5%; Appendix S2). However, the permutation importance of this climatic variable for *S. sponhemica* was zero. In contrast, the permutation importance was highest for both cytotypes for the variable mean temperature of the coldest month (*S. rosacea*: 47.0 and *S. sponhemica*: 56.6), which also had the second highest contribution to the model for *S. sponhemica* (14.8%) and the third highest for *S. rosacea* (9.2%; Appendix S2).

## DISCUSSION

The results of our study confirmed that both morphologically defined taxa have different ploidy levels (*S. sponhemica:* 6*x* = 48; *S. rosacea:* 8*x* = 64). Our chromosome numbers for *S. rosacea* are in agreement with those reported previously for the taxon by Philp (1934), and Catcheside and Heslop‐Harrison (reported in Webb, 1950) for material from Ireland. The 48 chromosomes found for *S. sponhemica* are within the range (46–52) reported in a study by Drábková (2000) using material from Czechia. In contrast to most other studies of species with different ploidy levels (Kolář et al., 2015), we found no mixed cytotype populations but complete spatial segregation of the two cytotypes.

There are at least three nonexclusive explanations for the complete spatial segregation of the cytotypes of *Saxifraga*. Minority cytotypes may become quickly excluded in mixed populations (Levin, 1975), cytotypes may have different ecological requirements (Ramsey, 2011; Kolář et al., 2015; McAllister et al., 2015; Muñoz‐Pajares et al., 2018), or historical factors such as differences in recolonization history and stochastic events may have shaped the distribution of the cytotypes (Sexton et al., 2009).

The minority cytotype exclusion hypothesis (Levin, 1975) predicts that if a different cytotype invades an established population of another cytotype, the reproductive success will be frequency dependent and the minority cytotype will be rapidly excluded from the population. In *Chamerion angustifolium*, Husband and Schemske (2000) found clear evidence that minority cytotype exclusion can operate in natural populations. Reproductive costs facing the minority cytotype may also explain the spatial segregation in *Ranunculus adoneus* (Baack, 2005). Infertility of hybrid 7*x* progeny resulting from crosses between 6*x* and 8*x Saxifraga* cytotypes might also have contributed to the spatial segregation of the two cytotypes in Central Europe.

However, several lines of evidence suggest that niche differentiation may be the main factor responsible for the spatial segregation. The WorldClim data, as well as the data from our local data loggers and the performance of the two cytotypes at the transplant site in Iceland, indicate that populations of *S. rosacea* can exist at much lower mean annual temperatures than those of *S. sponhemica*. The lower frost tolerance of *S. sponhemica* was corroborated by the results of the freezing experiment, in which the tolerance to severe frost of the hexaploid *S. sponhemica* from Central Europe was much lower than that of the octoploid *S. rosacea* from the same region. Conversely, reproductive performance of the populations of *S. sponhemica* in the warmer climate of the transplant site in Luxembourg was much higher than that of *S. rosacea* from the same region, and mean annual temperatures measured by data loggers placed in the Central European populations were higher in populations of *S. sponhemica* than in those of *S. rosacea*. There was some evidence of adaptation to regional conditions in *S. rosacea*. The subarctic populations of *S. rosacea* survived slightly better in Iceland than the plants from Central Europe, but were clearly maladapted to the warmer conditions in Luxembourg, where no plants survived over two years.

Other studies that investigated differences in environmental characteristics between populations of different cytotypes also found differences in climate (Nakagawa, 2006; Li et al., 2010; Manzaneda et al., 2012; Thompson et al., 2014; Muñoz‐Pajares et al., 2018), but this is not a general pattern (Godsoe et al., 2013; Glennon et al., 2014). Habitats of cytotypes may also differ in soil characteristics (Černá and Münzbergová, 2015) or be segregated by altitude (Husband and Schemske, 2000; Šafářová, 2011).

Although it has been stressed that niche differentiation between cytotypes should be ideally studied using reciprocal transplant experiments (Ramsey, 2011; Kolář et al., 2017), few studies have done so. Reciprocal transplant experiments with diploid and tetraploid *Anthoxanthum* species revealed local adaptation of the different cytotypes (Flegrová and Krahulec, 1999). Similarly, a study of *Chamerion angustifolium* cytotypes found that diploids and tetraploids survived best at their native elevations (Martin and Husband, 2013) and McIntyre and Strauss (2017) found evidence for local adaptation of cytotypes in *Claytonia perfoliata*. In contrast, other reciprocal transplant experiments failed to find clear evidence of local adaptation of cytotypes. For example, Buggs and Pannell (2007) found higher fitness for diploid than for hexaploid *Mercurialis annua* across all environments. In *Allium oleraceum*, plants of the local ploidy level showed higher performance only for some fitness traits (Duchoslav et al., 2017). For the 2*x*, 4*x*, and 6*x* species of the *Senecio carniolicus* complex, relative fitness of residents and transplants appear to depend on life‐history stage (Hülber et al., 2018; see also Raabová et al., 2008). Our experimental planting of rosettes from many populations of the two cytotypes of *Saxifraga* into two transplant sites with contrasting climates was not a reciprocal transplant experiment in the strict sense because the plants did not occur naturally at exactly those sites, but the results nevertheless suggest greater adaptation of *S. rosacea* to cold conditions and of *S. sponhemica* to warmer conditions.

Niche modeling suggested that both cytotypes of *Saxifraga* could co‐occur in many parts of Central Europe. Moreover, we found no differentiation in terms of accompanying vegetation or substrate composition between the two taxa in Central Europe. Both are rare species restricted to rock habitats where there is hardly any competition from other species. However, there are no contact zones between the cytotypes in central Europe. A possible reason is subtle differences in the climatic niches and the microhabitats, which are not reflected in the larger scale climatic data (WordClim and niche modeling), but are reflected in the higher mean annual temperatures measured locally with the data loggers in the Central European populations of *S. sponhemica* than in those of *S. rosacea*. Furthermore, historical factors may have affected the distributions. Both taxa are considered to be Ice Age relicts, which today are rare and restricted to isolated rock habitats, but may have been much more common during the Ice Age (Thorn, 1960; Walter and Straka, 1970). There could have been a large random component in the extinction of populations due to the invasion of trees after the Ice Age, contributing to the current pattern of distribution in Central Europe. Its greater frost tolerance may have allowed *S. rosacea* to colonize habitats in Scandinavia once they became available, while this appears not to have been possible for *S. sponhemica*.

Several authors have advanced the hypothesis that higher ploidy levels may be associated with a greater tolerance of extreme conditions and a greater niche breadth (e.g., Grant, 1981; Soltis et al., 2014), but results of studies of the relationship between niche breadth and ploidy level have been conflicting. In the genus *Clarkia*, polyploid species have significantly larger ranges than do diploid species (Lowry and Lester, 2006) and in *Claytonia perfoliata*, polyploids occupy distinct and broader niches relative to diploids (McIntyre, 2012). In contrast, no differences in mean range breadth were observed between diploid and polyploid congeners in a sample from diploid and polyploid species of North American angiosperms (Martin and Husband, 2009). In a study of the genus *Phalaris*, there was no general support for broader niche breadths of polyploids (Visser and Molofsky, 2015), and in *Primula*, climatic niches of polyploid species were narrower than those of diploid species (Theodoridis et al., 2013). In the Potentilleae tribe of the Rosaceae, transitions to higher ploidy are actually associated with reduced range size and abiotic breadth (Brittingham et al., 2018).

Our results support the hypothesis of increasing niche breadth with ploidy level because they suggest that the greater hardiness of the octoploid *S. rosacea* may explain why its range extends much farther to the north than that of the hexaploid *S. sponhemica*. The distribution of the closely related decaploid *S. cespitosa* (10*x* = 80) extends even farther to the north (up to 80°N). The three taxa may thus be an example of increasingly greater genetic flexibility to cope with the harsher conditions in the subarctic and arctic environments with increasing ploidy level (Brochmann et al., 2004; Rice et al., 2019). Tolerance of a large range of environmental conditions and a greater distributional range may be due to genetic differentiation among populations or due to phenotypic plasticity. Genetic differences in survival among populations were larger for the octoploid *S. rosacea* than for the hexaploid *S. sponhemica* at both transplant sites suggesting that greater genetic variability contributes to the larger distributional range of *S. rosacea*. The considerable variation found in the response of the different populations of the two cytotypes of *Saxifraga* to environmental conditions suggests that studies should not only consider the overall niche differentiation among cytotypes, but also genetic variation among populations within cytotypes.

In Central Europe, where both cytotypes of *Saxifraga* occur under broadly similar conditions, there were no differences in mean population characteristics except for a higher number of flowers per inflorescence in *S. rosacea*, which was, however, compensated by a lower number of inflorescences per cushion area. In contrast, plants in the Icelandic populations were much smaller and there were hardly any large plants. The very small size of adult *S. rosacea* plants in Iceland may indicate that this taxon is at its northern range limit in the subarctic. However, plants from Iceland also grew much slower than those from other populations when grown in Central Europe, were less plastic, and did not survive until the end of the experiment, suggesting adaptation to the subarctic conditions.

## CONCLUSIONS

Our results suggest that the different geographical distributions of the octoploid *S. rosacea* and the hexaploid *S. sponhemica* can at least be partially explained by the greater cold hardiness of *S. rosacea*. In the absence of strong habitat differences in Central Europe, reproductive isolation could explain why both taxa are not sympatric in Central Europe and why there are no known mixed populations where both taxa occur. To further clarify the taxonomic status of the two *Saxifraga rosacea* subspecies, molecular genetic studies and crossing experiments will be necessary. Our results support the hypothesis that niche differentiation between cytotypes can lead to spatial segregation, that higher ploidy levels may result in a broader ecological niche, and in particular, greater tolerance of more extreme conditions.

## AUTHOR CONTRIBUTIONS

G.C., D.M., L.D., N.E., and S.H. conceived the study and prepared the manuscript; G.C. and L.D. conducted field and laboratory work; D.M., G.C., and L.D. analyzed the data.

## Supporting information


**APPENDIX S1.** Locations of *Saxifraga rosacea* and *Saxifraga sponhemica*.

**APPENDIX S2.** Environmental niche modeling with MaxEnt.

## Data Availability

Data are available at Mendeley at https://data.mendeley.com/datasets/jvwmfvyjcr/1.
