# Supplementary material for: Ecological niche differences between two polyploid cytotypes of Saxifraga rosacea
Source: Am J Bot. 2020 Feb 17;107(3):423–35. doi: 10.1002/ajb2.1431 (PMC7216898; doi:10.1002/ajb2.1431)
Supplement: Supplementary file 1 — APPENDIX S1. Locations of Saxifraga rosacea and Saxifraga sponhemica. [file AJB2-107-423-s001.docx]

Decanter et al.—*American Journal of Botany* 2020– Appendix S1

**Appendix S1.** Locations of *Saxifraga rosacea* and *Saxifraga sponhemica*

| **Country** | **Locality of *Saxifraga rosacea*** | **latwgs84** | **lonwgs84** | **Collector** |
| --- | --- | --- | --- | --- |
| France | Beaumes-les-Messieurs, Reculée de Beaumes | 46.68957 | 5.63775 | Tania Walisch |
| France | Beaumes-les-Messieurs, Bois de Saint-Aldegrin | 46.69697 | 5.65441 | Tania Walisch |
| France | Hartmannswillerkopf | 47.86003 | 7.16583 | Nora Elvinger |
| Germany | MTB 4814: Lennestadt (North Rhine-Westphalia) | 51.174912 | 8.041437 | GBIF - FlorKart BfN |
| Germany | Bodensteiner Ley near Runkel (North Rhine-Westphalia) | 50.39192 | 8.17723 | Nora Elvinger |
| Germany | MTB 5615: Villmar (Hesse) | 50.374899 | 8.20811 | GBIF - FlorKart BfN |
| Germany | MTB 8315: Waldshut-Tiengen (Baden-Württemberg) | 47.67488 | 8.29145 | GBIF - FlorKart BfN |
| Germany | Bad Laasphe (Hesse) | 50.9278306 | 8.425361 | Nora Elvinger |
| Germany | MTB 5016: Bad Laasphe (North Rhine-Westphalia) | 50.924909 | 8.458118 | GBIF - FlorKart BfN |
| Germany | MTB 5017: Biedenkopf (Hesse) | 50.974918 | 8.541454 | GBIF - FlorKart BfN |
| Germany | MTB 4917: Battenberg (Eder) (Hesse) | 51.024918 | 8.624786 | GBIF - FlorKart BfN |
| Germany | MTB 4918: Frankenberg (Eder) (Hesse) | 51.024918 | 8.708124 | GBIF - FlorKart BfN |
| Germany | MTB 6120: Obernburg am Main (Bavaria) | 49.824909 | 9.041475 | GBIF - FlorKart BfN |
| Ireland | Caher Connel (County Clare) | 53.04072 | 9.13926 | Nora Elvinger |
| Germany | MTB 7821: Veringenstadt (Baden-Württemberg) | 48.174896 | 9.208142 | GBIF - FlorKart BfN |
| Germany | MTB 7721: Gammertingen (Baden-Württemberg) | 48.224893 | 9.208143 | GBIF - FlorKart BfN |
| Germany | Hermentingen (Baden-Württemberg) | 48.19618 | 9.22099 | Nora Elvinger |
| Ireland | Black Head (County Clare) | 53.15351 | 9.26426 | Nora Elvinger |
| Germany | MTB 7521: Reutlingen (Baden-Württemberg) | 48.474896 | 9.291479 | GBIF - FlorKart BfN |
| Germany | MTB 4822: Gudensberg (Hesse) | 51.174922 | 9.37481 | GBIF - FlorKart BfN |
| Germany | MTB 7522: Bad Urach (Baden-Württemberg) | 48.474899 | 9.458154 | GBIF - FlorKart BfN |
| Germany | MTB 7623: Mehrstetten (Baden-Württemberg) | 48.324899 | 9.624825 | GBIF - FlorKart BfN |
| Germany | MTB 7624: Schelklingen (Baden-Württemberg) | 48.324902 | 9.708157 | GBIF - FlorKart BfN |
| Germany | MTB 7524: Blaubeuren (Baden-Württemberg) | 48.424902 | 9.70816 | GBIF - FlorKart BfN |
| Ireland | Macgillycuddy Reeks (County Kerry) | 52.00291 | 9.75764 | Nora Elvinger |
| Germany | MTB 7324: Geislingen an der Steige-West (Baden-Württemberg) | 48.674899 | 9.791494 | GBIF - FlorKart BfN |
| Germany | MTB 7225: Heubach (Baden-Württemberg) | 48.774909 | 9.874834 | GBIF - FlorKart BfN |
| Germany | MTB 7525: Ulm-Nordwest (Baden-Württemberg) | 48.424902 | 9.958165 | GBIF - FlorKart BfN |
| Germany | Wentalweible (Baden-Württemberg) | 48.71369 | 10.016 | Nora Elvinger |
| Germany | Wental (Baden-Württemberg) | 48.73406 | 10.01634 | Nora Elvinger |
| Germany | Bernstadt (Baden-Württemberg) | 48.51801 | 10.03513 | Nora Elvinger |
| Germany | MTB 7526: Ulm Nordost (Baden-Württemberg) | 48.474906 | 10.041501 | GBIF - FlorKart BfN |
| Germany | MTB 7226: Oberkochen (Baden-Württemberg) | 48.724915 | 10.041502 | GBIF - FlorKart BfN |
| Germany | MTB 7426: Langenau (Baden-Württemberg) | 48.524909 | 10.041504 | GBIF - FlorKart BfN |
| Germany | Königsbronn (Baden-Württemberg) | 48.73524 | 10.11465 | Nora Elvinger |
| Germany | MTB 7326: Heidenheim an der Brenz (Bavaria) | 48.624915 | 10.124836 | GBIF - FlorKart BfN |
| Germany | Eselsburgertal (Baden-Württemberg) | 48.6082077 | 10.1784619 | Nora Elvinger |
| Germany | MTB 7327: Giengen an der Brenz (Baden-Württemberg) | 48.624909 | 10.20817 | GBIF - FlorKart BfN |
| Germany | MTB 7227: Neresheim-West (Baden-Württemberg) | 48.774915 | 10.208174 | GBIF - FlorKart BfN |
| Ireland | Mount Brandon (County Kerry) | 52.23679 | 10.25107 | Nora Elvinger |
| Germany | MTB 8527: Oberstdorf (Bavaria) | 47.474896 | 10.291515 | GBIF - FlorKart BfN |
| Germany | MTB 5328: Wasungen (Thuringia) | 50.624931 | 10.374845 | GBIF - FlorKart BfN |
| Germany | MTB 7228: Neresheim-Ost (Baden-Württemberg) | 48.774918 | 10.374847 | GBIF - FlorKart BfN |
| Germany | MTB 4229: Braunlage (Lower Saxony) | 51.774947 | 10.541514 | GBIF - FlorKart BfN |
| Germany | MTB 4129: Bad Harzburg (Lower Saxony) | 51.824944 | 10.624846 | GBIF - FlorKart BfN |
| Germany | MTB 4230: Elbingerode im Harz (Saxony-Anhalt) | 51.72495 | 10.708186 | GBIF - FlorKart BfN |
| Germany | MTB 4330: Benneckenstein im Harz (Saxony-Anhalt) | 51.674947 | 10.791521 | GBIF - FlorKart BfN |
| Germany | MTB 4130: Wernigerode (Saxony-Anhalt) | 51.82495 | 10.791523 | GBIF - FlorKart BfN |
| Germany | Rübeland, Hermannshöhlen (Saxony-Anhalt) | 51.75403 | 10.8366 | Nora Elvinger |
| Germany | Rübeland (Saxony-Anhalt) | 51.75315 | 10.84278 | Nora Elvinger |
| Germany | Neuwerk (Saxony-Anhalt) | 51.75759 | 10.86583 | Nora Elvinger |
| Germany | MTB 4231: Blankenburg (Saxony-Anhalt) | 51.77495 | 10.874858 | GBIF - FlorKart BfN |
| Germany | MTB 4331: Hasselfelde (Saxony-Anhalt) | 51.674947 | 10.958192 | GBIF - FlorKart BfN |
| Germany | MTB 4232: Quedlinburg (Saxony-Anhalt) | 51.724954 | 11.041526 | GBIF - FlorKart BfN |
| Germany | MTB 4132: Halberstadt (Saxony-Anhalt) | 51.82495 | 11.041526 | GBIF - FlorKart BfN |
| Germany | MTB 7932: Utting am Ammersee (Bavaria) | 48.074912 | 11.041536 | GBIF - FlorKart BfN |
| Germany | MTB 4332: Harzgerode (Saxony-Anhalt) | 51.62495 | 11.124862 | GBIF - FlorKart BfN |
| Germany | MTB 5232: Stadtilm (Thuringia) | 50.724941 | 11.124866 | GBIF - FlorKart BfN |
| Germany | MTB 6133: Muggendorf (Bavaria) | 49.874934 | 11.208208 | GBIF - FlorKart BfN |
| Germany | MTB 4333: Pansfelde (Saxony-Anhalt) | 51.674947 | 11.291538 | GBIF - FlorKart BfN |
| Germany | MTB 6233: Ebermannstadt (Bavaria) | 49.774928 | 11.291539 | GBIF - FlorKart BfN |
| Germany | MTB 6033: Hollfeld (Bavaria) | 49.974938 | 11.29154 | GBIF - FlorKart BfN |
| Germany | MTB 6133: Muggendorf (Bavaria) | 49.824931 | 11.291542 | GBIF - FlorKart BfN |
| Germany | MTB 7034: Kipfenberg (Bavaria) | 48.974922 | 11.374874 | GBIF - FlorKart BfN |
| Germany | MTB 6334: Betzenstein (Bavaria) | 49.624931 | 11.374874 | GBIF - FlorKart BfN |
| Germany | Kinding (Bavaria) | 48.98774 | 11.43491 | Nora Elvinger |
| Germany | MTB 5434: Leutenberg (Thuringia) | 50.574944 | 11.458207 | GBIF - FlorKart BfN |
| Germany | MTB 6434: Hersbruck (Bavaria) | 49.524928 | 11.458209 | GBIF - FlorKart BfN |
| Germany | MTB 6234: Pottenstein (Bavaria) | 49.724934 | 11.45821 | GBIF - FlorKart BfN |
| Germany | MTB 5334: Saalfeld a.d. Saale (Thuringia) | 50.624938 | 11.45821 | GBIF - FlorKart BfN |
| Germany | MTB 6934: Beilngries (Bavaria) | 49.024925 | 11.458213 | GBIF - FlorKart BfN |
| Germany | Bronn (Bavaria) | 49.72732 | 11.46012 | Nora Elvinger |
| Germany | Hersbrück (Bavaria) | 49.50737 | 11.49935 | Nora Elvinger |
| Germany | Velden (Bavaria) | 49.6097 | 11.51539 | Nora Elvinger |
| Germany | Pfaffenhofen (Bavaria) | 49.63659 | 11.53114 | Nora Elvinger |
| Germany | MTB 5335: Ziegenrück (Thuringia) | 50.624944 | 11.541543 | GBIF - FlorKart BfN |
| Germany | MTB 6235: Pegnitz (Bavaria) | 49.724938 | 11.541544 | GBIF - FlorKart BfN |
| Germany | MTB 6135: Creuβen (Bavaria) | 49.874938 | 11.541547 | GBIF - FlorKart BfN |
| Germany | MTB 5435: Liebengrün (Thuringia) | 50.574947 | 11.541547 | GBIF - FlorKart BfN |
| Germany | MTB 6335: Auerbach in der Oberpfalz (Bavaria) | 49.624934 | 11.541547 | GBIF - FlorKart BfN |
| Germany | MTB 6935: Dietfurt an der Altmühl (Bavaria) | 49.074928 | 11.54155 | GBIF - FlorKart BfN |
| Germany | Seeweiher (Bavaria) | 49.69057 | 11.54637 | Nora Elvinger |
| Germany | Auerbach-Michelfeld (Bavaria) | 49.696114 | 11.546667 | observation |
| Germany | Michelfeld (Bavaria) | 49.62971 | 11.54772 | Nora Elvinger |
| Germany | Dietfurt (Bavaria) | 49.05828 | 11.57027 | Nora Elvinger |
| Germany | Drognitz (Thuringia) | 50.59522 | 11.58729 | Nora Elvinger |
| Germany | MTB 7035: Schamhaupten (Bavaria) | 48.974931 | 11.624885 | GBIF - FlorKart BfN |
| Germany | MTB 6435: Pommelsbrunn (Bavaria) | 49.524934 | 11.624886 | GBIF - FlorKart BfN |
| Germany | MTB 5536: Hirschberg an der Saale (Thuringia) | 50.474941 | 11.708214 | GBIF - FlorKart BfN |
| Germany | MTB 5436: Schleiz (Thuringia) | 50.524941 | 11.708218 | GBIF - FlorKart BfN |
| Germany | Gräfenwarth (Thuringia) | 50.52518 | 11.71307 | Nora Elvinger |
| Germany | Pöritzsch (Thuringia) | 50.49714 | 11.72978 | Nora Elvinger |
| Germany | MTB 5737: Schwarzenbach an der Saale (Bavaria) | 50.274947 | 11.958223 | GBIF - FlorKart BfN |
| Germany | Fichtelgebirge nature parc (Bavaria) | 50.063309 | 11.962051 | observation |
| Germany | Wojaleite national protection zone (Bavaria) | 50.253891 | 11.972593 | observation |
| Germany | MTB 5138: Gera (Thuringia) | 50.82495 | 12.041558 | GBIF - FlorKart BfN |
| Germany | MTB 5238: Weida (Thuringia) | 50.774947 | 12.041558 | GBIF - FlorKart BfN |
| Germany | MTB 5638: Bobenneukirchen (Saxony) | 50.324947 | 12.04156 | GBIF - FlorKart BfN |
| Germany | MTB 5438: Plauen (Saxony) | 50.57495 | 12.124901 | GBIF - FlorKart BfN |
| Germany | Meschwitz (Saxony) | 50.54024 | 12.16931 | Nora Elvinger |
| Germany | Reschwitzmühle (Saxony) | 50.551675 | 12.1754167 | Nora Elvinger |
| Germany | MTB 5439: Treuen (Saxony) | 50.524947 | 12.208238 | GBIF - FlorKart BfN |
| Germany | MTB 5343: Geyer (Saxony) | 50.67496 | 12.87492 | GBIF - FlorKart BfN |
| Germany | MTB 4945: Roβwein (Saxony) | 51.07497 | 13.2916 | GBIF - FlorKart BfN |
| Germany | MTB 5147: Frauenstein (Saxony) | 50.82497 | 13.541606 | GBIF - FlorKart BfN |
| Germany | MTB 5050: Königstein (Saxony) | 50.974973 | 14.124958 | GBIF - FlorKart BfN |
| Germany | MTB 4553: Nochten (Saxony) | 51.474986 | 14.541635 | GBIF - FlorKart BfN |
| Germany | MTB 4855: Gorlitz (Saxony) | 51.124986 | 14.958315 | GBIF - FlorKart BfN |
| Iceland | Adaldalsrhaun | 65.93488 | -17.49859 | Nora Elvinger |
| Iceland | after Ólafsfjördur | 65.95189 | -18.83559 | Nora Elvinger |
| Iceland | Austuràrgil Aðalbòlsheiði | 65.09406 | -20.61188 | Hörður Kristinsson |
| Iceland | Böðvarsdalur Vopnafjörður | 65.754 | -14.537 | Eypor Einarsson |
| Iceland | Eldgjà | 63.946 | -18.646 | Eypor Einarsson |
| Iceland | Fagurhòlsmyri Öraefum | 63.877 | -16.648 | Bergpor Johannsson 2005 |
| Iceland | Fljòtshlíð | 63.723 | -19.95 | Bergpor Johannsson 2005 |
| Iceland | Geirmundarstadir | 65.78751 | -21.75386 | Nora Elvinger |
| Iceland | Grindavík | 63.841 | -22.429 | Bergpor Johannsson 2005 |
| Iceland | Herdísarvík Reykjanesskaga | 63.869 | -21.814 | Eypor Einarsson |
| Iceland | Heydalsà Steingrímsfjörður | 65.636 | -21.535 | Eypor Einarsson |
| Iceland | Hörgshlíð Mjòafjörður | 65.844 | -22.604 | Eypor Einarsson |
| Iceland | Hrunamannahreppi | 64.258 | -20.235 | Eypor Einarsson |
| Iceland | Keilir Reykjanesi | 63.941 | -22.161 | Bergpor Johannsson 2005 |
| Iceland | Kròkamyri Reykjanesskaga | 63.901 | -22.104 | Kristbjörn Egilsson |
| Iceland | Maelifellsdalur Skagafjörður | 65.37991 | -19.3974 | Hörður Kristinsson |
| Iceland | Mjòlkà, Arnarfjörður | 65.774 | -23.159 | Bergpor Johannsson 2005 |
| Iceland | near Egilsstadir | 65.25416 | -14.42315 | Nora Elvinger |
| Iceland | near road 515 | 64.6206 | -21.36601 | Nora Elvinger |
| Iceland | near Svartifoss | 64.02471 | -16.94057 | Nora Elvinger |
| Iceland | on road 1 close to Laufskálavarða (Kir) | 63.62068 | -18.44808 | Nora Elvinger |
| Iceland | on road 427 | 63.85175 | -22.20126 | Nora Elvinger |
| Iceland | on road 43 to Grindavik | 63.88741 | -22.40578 | Nora Elvinger |
| Iceland | on road 43 to Grindavik | 63.88518 | -22.41832 | Nora Elvinger |
| Iceland | Öxi | 64.81394 | -14.62265 | Nora Elvinger |
| Iceland | Raudaberg | 64.3499 | -15.67652 | Nora Elvinger |
| Iceland | Reykjanes Reykjanesi | 63.817 | -22.698 | Bergpor Johannsson 2005 |
| Iceland | Seljafoss | 63.61587 | -19.98787 | Nora Elvinger |
| Iceland | Seljalandsfoss | 63.614 | -19.998 | Bergpor Johannsson 2005 |
| Iceland | Skaftafell Vatnajökull National Park | 64.01318 | -16.96764 | Nora Elvinger |
| Iceland | Skeljabrekka Borgarfjörður | 64.538 | -21.749 | Eypor Einarsson |
| Iceland | Skógafoss | 63.53151 | -19.5122 | Nora Elvinger |
| Iceland | Skógafoss | 63.531 | -19.531 | Bergpor Johannsson 2005 |
| Iceland | Skògar undir Eyjafjallajökull | 63.525 | -19.493 | Bergpor Johannsson 2005 |
| Iceland | Spillir Sùgandafjörður | 66.127 | -23.546 | Bergpor Johannsson 2005 |
| Iceland | Torfnafjall Fljótum | 66.12657 | -19.03662 | Hörður Kristinsson |
| Iceland | Vìk | 63.42259 | -19.01556 | Nora Elvinger |
| Iceland | Vík Myrdal | 63.42 | -19.026 | Bergpor Johannsson 2005 |
| Iceland | Vogastapi Reykjanesi | 63.97 | -22.443 | Eypor Einarsson |
| Sweden | Sm. Jönköping. Fäng tr. Jönköpings Simsällskap | 57.776933 | 14.15806 | Eriksson, Erik |
| Sweden | Sm. Jönköping. | 57.781999 | 14.159527 | Eriksson, Eskil |
| Sweden | Ekenhaga | 57.184036 | 14.048284 | Petersson, Bernhard |
| Sweden | Småland, Berget. | 56.805334 | 14.037221 | Christoffersson, Judith |
| Sweden | Hools prästgård. | 57.974568 | 12.664744 | Åberg, Sven |
| Sweden | Göteborg Slottsskogen | 57.685899 | 11.938741 | Johansson, Yngve |
| Sweden | Halland. Ränneslöv s:n, Ålstorp. Förvildad utanför en trädgård. | 56.460745 | 13.152915 | Johansson, Yngve |
| Sweden | Lindsdal, Lidhemsväg, ödehus vid IP. Förvildad trädgård | 56.727111 | 16.295299 | Davidsson, Sven |
| Norway | Salhus i havets nivaa | 60.50349 | 5.26739 | Ivar Jørstad, Finn Wischmann |
| Norway | Indplantet i Tøien bot. have fra Hovlandsfjeld paa Modum";" Petrus. | 59.91708 | 10.77061 | N. Moe, Johannes Lid scr. |
| Norway | Lørenskog Låsby, langs veien | 59.89159 | 10.98375 | Halfdan Rui |
| Norway | Stavanger: Vølstadveien, N-siden av Lt. Stokkavatn. | 58.97285 | 5.68682 | John Inge Johnsen |
| Norway | Espa stasjon, vegkant langs vegen opp til nåvaerende E6 | 60.58123 | 11.27083 | Anders Often, Tore Berg, Reidar Haugan |
| Norway | Gimle | 58.16303 | 8.00435 | Torleiv Hannaas, Finn Wischmann |
| Norway | Ved Malms stue, Vik | 65.31047 | 12.17789 | Knut Strompdal |
| Norway | Vandeskog, Sveio. Hage. | 59.53806 | 5.28189 | Olav Vandeskog |
| Norway | Salhus ved Bergen | 60.50349 | 5.26739 | Trygve Slettebakken |
| Norway | Etne Kambo Have | 59.67501 | 5.95618 | Chr. Sommerfelt, Finn Wischmann |
| Norway | Fra fru Strømsøs have [paa] Aasvei - Trondhjem | 63.41893 | 10.37213 | Einar Fondal |
| Norway | Byaasen, | 63.41005 | 10.36169 | Einar Fondal |
| Norway | Salhus ved Bergen | 60.50349 | 5.26739 | Trygve Slettebakken |
| Norway | Udsigten, Aasveien | 63.42341 | 10.37234 | Einar Fondal |
| Norway | Hjartland, ytre Strandberg | 66.05357 | 12.85024 | Tommy Prestø |
| Norway | Pl. i steinbed hos frk. Sellaeg. Jonsv.v. | 63.42302 | 10.4124 | Einar Fondal |
| Finland | Svisskärsfjärden, Kristinestad | 62.248368 | 21.363466 | Finnish Museum of Natural History Collections |
| Finland | Villinki, Helsingfors | 60.155933 | 25.125525 | Finnish Museum of Natural History Collections |
| Finland | Hiidenvesi | 60.331021 | 24.145236 | Finnish Museum of Natural History Collections |
| Finland | Nurmijärvi | 60.502349 | 24.639979 | Finnish Museum of Natural History Collections |
| Finland | Virolahti | 60.531059 | 27.55244 | Finnish Museum of Natural History Collections |
| Norway | Hinnøya: Kanbeogen, mellom Damveien og Kanebogelva ved trafostasjonen. | 68.77104 | 16.54431 | Torbjørn Alm, Unni Bjerke Gamst |
| Norway | Vefsn: Knausen i Halsøy. Steinbedd. | 65.86467 | 13.20262 | Peter Benum |
| Norway | Tverlandet naturreservat | 67.270391 | 14.720531 | Grete Nytrøen Kvavik, Mats Nettelbladt, Ingvild Gabrielsen, Gunnar Rofstad |
| Norway | Fana, Paradis. | 60.3366 | 5.346 | G. F. Heiberg, R. Elven |
| Norway | Fredrikshald, | 59.13014 | 11.39395 | E. Ryan scr., Sigmund Sivertsen |
| Sweden | Rinkaby | 55.984255 | 14.269795 | Knut Andersson |
| Sweden | Karlstad | 59.381584 | 13.505989 | Oskar Wer |
| Norway | Svorkmo, 'Austli' v. Aspøl | 63.18386 | 9.74338 | Kjetil Bevanger |
| Norway | Byåsveien | 63.41902 | 10.36212 | Arne Lie |
| Norway | Pl. i steinbed hos fru dommer Eriksen | 63.42322 | 10.39237 | Einar Fondal |
| Norway | Austbygda kirke | 60.02576 | 8.82806 | Elisabeth Bodvin, Jan-Henrik Bodvin, Reidar Elven |
| Norway | Ved Eg | 58.16276 | 7.97037 | John Nuland |
| Sweden | Vetlanda N | 57.48913 | 15.3375 | T. Merkert |
| Sweden | Växjö Ö | 56.86252 | 15.08008 | Curt Mossberg |
| Sweden | Bäckseda, Vetlanda | 57.400563 | 15.076892 | Alf Lindström |
| Sweden | Aneby Ö | 57.80614 | 14.99194 | Jörgen Josefsson |
| Sweden | Alvesta C | 56.84864 | 14.531 | Åke Widgren |

| **Country** | **Locality of *Saxifraga sponhemica*** | **latwgs84** | **lonwgs84** | **Collector** |
| --- | --- | --- | --- | --- |
| Belgium | Bouillon castle lower part | 49.79275 | 5.063833 | Tania Walisch |
| Belgium | Bouillon, Bastion de Bretagne | 49.796944 | 5.069527 | Tania Walisch |
| Belgium | Robertville | 50.452566 | 6.1025777 | Nora Elvinger |
| Czech Republic | Blešno | 50.48203 | 13.90565 | Tania Walisch |
| Czech Republic | Děkovka | 50.49008 | 13.92368 | Tania Walisch |
| Czech Republic | Ostry | 50.5318 | 13.95135 | Tania Walisch |
| Czech Republic | Boreč 1 | 50.5151 | 13.98975 | Tania Walisch |
| Czech Republic | Tetinske Skaty | 49.57007 | 14.06241 | Tania Walisch |
| Czech Republic | Voškov 2 | 49.920555 | 14.183027 | Tania Walisch |
| Czech Republic | Voškov 1 | 49.917944 | 14.197166 | Tania Walisch |
| France | Planches/Arbois | 46.879066 | 5.812994 | Tania Walisch |
| France | Salins | 46.926122 | 5.918511 | Tania Walisch |
| Germany | Mettendorf (Rhineland-Palatinate) | 49.924877 | 6.20805 | GBIF - FlorKart BfN |
| Germany | Mürlenbach (Rhineland-Palatinate) | 50.174893 | 6.624731 | GBIF - FlorKart BfN |
| Germany | Gerolstein (Rhineland-Palatinate) | 50.19539 | 6.62792 | Nora Elvinger |
| Germany | Lebach (Saarland) | 49.474883 | 6.874738 | GBIF - FlorKart BfN |
| Germany | Sohren (Rhineland-Palatinate) | 49.92489 | 7.208082 | GBIF - FlorKart BfN |
| Germany | Frauenburg | 49.666912 | 7.282493 | Tania Walisch |
| Germany | Naheloreleifels | 49.679854 | 7.288151 | Tania Walisch |
| Germany | Hammerstein crossing | 49.690271 | 7.289085 | Tania Walisch |
| Germany | Birkenfeld (Rhineland-Palatinate) | 49.674896 | 7.291417 | GBIF - FlorKart BfN |
| Germany | Hammerstein road | 49.687337 | 7.29939 | Tania Walisch |
| Germany | Baumholder (Rhineland-Palatinate) | 49.674896 | 7.374754 | GBIF - FlorKart BfN |
| Germany | Gemünden (Rhineland-Palatinate) | 49.82489 | 7.374755 | GBIF - FlorKart BfN |
| Germany | Bockenau (Rhineland-Palatinate) | 49.49656 | 7.40896 | Nora Elvinger |
| Germany | Kirn (Rhineland-Palatinate) | 49.774896 | 7.45809 | GBIF - FlorKart BfN |
| Germany | Norheim (Rhineland-Palatinate) | 49.4818 | 7.48537 | Nora Elvinger |
| Germany | Hellberg im Nahebergland (Rhineland-Palatinate) | 49.79314 | 7.495806 | GBIF - naturgucker |
| Germany | Waldböckelheim (Rhineland-Palatinate) | 49.824893 | 7.791434 | GBIF - FlorKart BfN |
| Germany | Dannenfels (Rhineland-Palatinate) | 49.624893 | 7.874766 | GBIF - FlorKart BfN |
| Luxembourg | Kautenbach, railroad crossing | 49.95164 | 6.016199 | Tania Walisch |
| Luxembourg | Kautenbach, Hockslay | 49.945055 | 6.026922 | Tania Walisch |
| Luxembourg | Unterschlinder (rock) | 49.921747 | 6.071765 | Tania Walisch |
| Luxembourg | Erpeldange 1 (scree) | 49.894458 | 6.115189 | Tania Walisch |
| Luxembourg | Vianden (parking) | 49.934721 | 6.198395 | Tania Walisch |
| Luxembourg | Vianden (castle wall) | 49.933399 | 6.207622 | Tania Walisch |
| Luxembourg | Bettel, Bicycle road | 49.922687 | 6.218112 | Tania Walisch |
| Luxembourg | between Vianden and Bettel | 49.922849 | 6.219157 | Tania Walisch |
| Luxembourg | Vianden, Roth | 49.928734 | 6.225124 | Tania Walisch |
