# Supplementary material for: Ecological niche differences between two polyploid cytotypes of Saxifraga rosacea
Source: Am J Bot. 2020 Feb 17;107(3):423–35. doi: 10.1002/ajb2.1431 (PMC7216898; doi:10.1002/ajb2.1431)
Supplement: Supplementary file 2 — APPENDIX S2. Environmental niche modeling with MaxEnt. [file AJB2-107-423-s002.docx]

Decanter et al.—*American Journal of Botany* 2020– Appendix S2

**Appendix S2.** Environmental niche modelling with MaxEnt.

All 19 bioclimatic variables available from the WorldClim v2.0 database (Fick and Hijmans, 2017) in a grid size of 0.86 km² (30 arc s) were used for tests run separately for *Saxifraga sponhemica* and *S. rosacea*. Based on the relative contribution of each bioclimatic variable to the final model the following variables were selected for the niche modelling: mean diurnal range, temperature seasonality, minimum temperature of the coldest month, temperature annual range, mean temperature of wettest quarter, mean temperature of driest quarter, mean temperature of warmest quarter, mean temperature of the coldest quarter, precipitation of wettest month, and precipitation of the warmest quarter.

***Analysis of variable contributions***

a) *Saxifraga sponhemica*

| **Variable** | **Percent contribution** | **Permutation importance** |
| --- | --- | --- |
| Temperature annual range | 41.5 | 0 |
| Min temperature of coldest month | 14.8 | 56.6 |
| Mean temperature of coldest quarter | 8.9 | 9.6 |
| Precipitation of warmest quarter | 7.9 | 2.9 |
| Mean temperature of driest quarter | 7.9 | 2.2 |
| Temperature seasonality | 7.7 | 3.2 |
| Mean diurnal Range | 4.9 | 3.7 |
| Mean temperature of warmest quarter | 3.2 | 17.9 |
| Precipitation of wettest month | 2.6 | 3.1 |
| Mean Temperature of Wettest Quarter | 0.6 | 0.9 |

b) *Saxifraga rosacea*

| **Variable** | | **Percent contribution** | **Permutation importance** |
| --- | --- | --- | --- |
| Temperature annual range | 49.4 | | 13.1 |
| Mean temperature of coldest quarter | 19.1 | | 25.2 |
| Min temperature of coldest month | 9.2 | | 47 |
| Mean temperature of driest quarter | 8.4 | | 3.1 |
| Mean diurnal range | 5.6 | | 0.7 |
| Precipitation of wettest month | 3 | | 2 |
| Mean temperature of warmest quarter | 2.9 | | 3 |
| Mean temperature of wettest quarter | 1.8 | | 0.6 |
| Precipitation of warmest quarter | 0.4 | | 1.1 |
| Temperature seasonality | 0.3 | | 4.1 |

The tables (a) and (b) give estimates of relative contributions of the WorldClim variables to the Maxent models. To determine the first estimate, in each iteration of the training algorithm, the increase in regularized gain is added to the contribution of the corresponding variable, or subtracted from it if the change to the absolute value of lambda is negative. For the second estimate, for each environmental variable in turn, the values of that variable on training presence and background data are randomly permuted. The model is reevaluated on the permuted data, and the resulting drop in training AUC is shown in the table, normalized to percentages. Values shown are averages over replicate runs.

Of all climatic variables in the Maxent model, for *S. sponhemica* the estimate of relative importance was highest for BIO6 (56.6), and for *S. rosacea* BIO7 contributed most (49.4%) to the model.


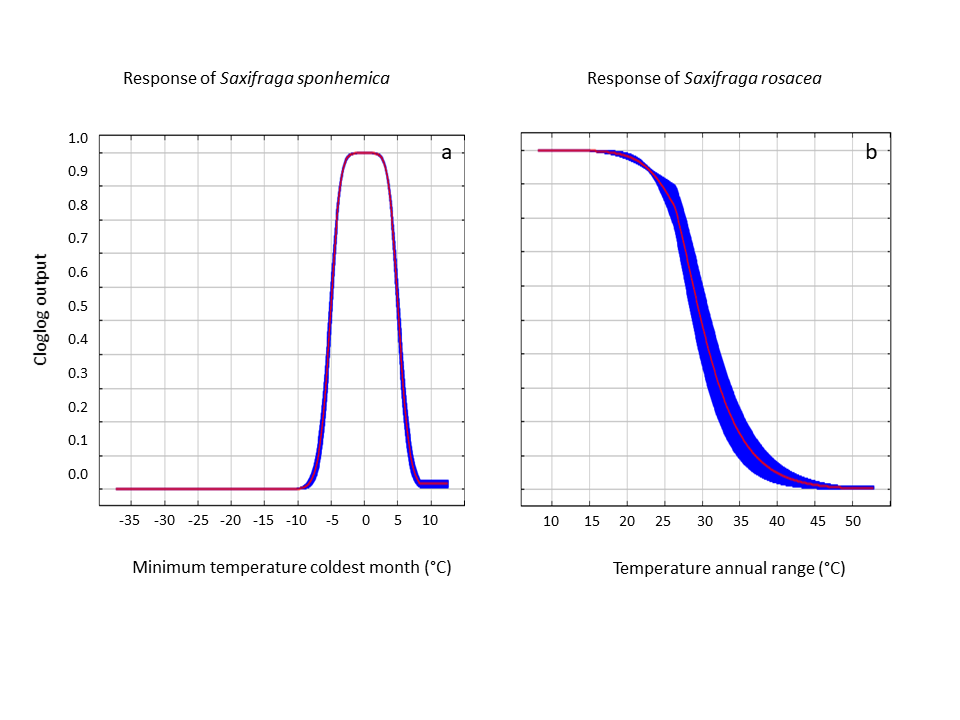


Legend to figure: Predicted probability of (a) the presence of *Saxifraga sponhemica* in relation to the minimum temperature of the coldest month (BIO6), and (b) the presence of *Saxifraga rosacea* in relation to temperature annual range (BIO7). The curves show the mean response of the 10 replicate Maxent runs (red) +/– 1 standard deviation (blue shade).

**LITERATURE CITED**

Fick, S. E., and R. J. Hijmans. 2017. WorldClim 2: new 1-km spatial resolution climate surfaces for global land areas: New climate surfaces for global land areas. *International Journal of Climatology* 37: 4302–4315.
